# Cytomegalovirus colitis in hospitalized inflammatory bowel disease patients in Taiwan: a referral center study

**DOI:** 10.1186/s12876-017-0586-9

**Published:** 2017-02-13

**Authors:** Meng-Tzu Weng, Chien-Chih Tung, Yi-Shuan Lee, Yew-Loong Leong, Ming-Jium Shieh, Chia-Tung Shun, Cheng-Yi Wang, Jau-Min Wong, Shu-Chen Wei

**Affiliations:** 1 Department of Internal Medicine, National Taiwan University Hospital and College of Medicine, No. 7 Chung-Shan South Road, Taipei City, Taiwan; 2 Department of Internal Medicine, Far Eastern Memorial Hospital, New Taipei City, Taiwan; 3 Department of Chemical Engineering & Materials Science, Yuan-Ze University, Taoyuan, Taiwan; 4 Department of Pathology and Forensic Medicine, National Taiwan University Hospital and College of Medicine, Taipei City, Taiwan; 5 Department of Internal Medicine, West Garden Hospital, Taipei City, Taiwan; 6 Department of Oncology, National Taiwan University Hospital and College of Medicine, Taipei City, Taiwan

**Keywords:** Inflammatory bowel disease, Crohn’s disease, Ulcerative colitis, Cytomegalovirus colitis

## Abstract

**Background:**

Colitis is exacerbated in patients with concurrent cytomegalovirus (CMV) infection and inflammatory bowel disease (IBD). We assessed the prevalence and clinical features of CMV colitis in hospitalized IBD patients.

**Methods:**

A retrospective study reviewed the data from January 1, 1998 through December 31, 2013 compiled at the National Taiwan University Hospital. The CMV colitis patients’ demographic data, clinical information, treatment regimens, pathologic findings, and outcome were analyzed.

**Results:**

A total of 673 IBD patients were hospitalized during the study period. There were 312 patients diagnosed with Crohn’s disease (CD) and 361 with ulcerative colitis (UC). CMV colitis was diagnosed as having positive inclusion bodies in colonic tissue. Six of the 312 CD patients (1.9%) and five of the 361 UC patients (1.4%) were diagnosed with CMV colitis. Compared to CD patients without CMV colitis, patients with CMV colitis were more often older (*p* < 0.005). Higher steroid usage was noted in the CMV positive group compared to age and gender matched CMV negative IBD patients (81.8% vs. 51.5%). Eight patients received ganciclovir treatment. Three patients who did not receive antiviral treatment had colitis flare-ups after the index admission.

**Conclusions:**

The prevalence of CMV colitis in hospitalized IBD inpatients was 1.6% in Taiwan. Two associated factors for CMV colitis in hospitalized IBD patients were that they were elderly in CD and were on higher doses of steroids. Routine histopathology studies and/or PCR for refractory colitis patients are suggested to diagnose CMV colitis. Once the diagnosis is made, antiviral treatment is recommended to decrease the colitis relapse rate.

## Background

Cytomegalovirus (CMV) infection is common and its prevalence has been reported from 40 to 100% in adults [1]. Primary CMV is generally asymptomatic or presents as a mild mononucleosis-like syndrome in healthy people [2]. The viremic phase is often self-limited and is followed by a lifelong latent phase [3]. Reactivation from latency can lead to a serious disease in immunocompromised individuals [4]. A distinction between CMV infection, meaning the positive detection of CMV by serology tests or PCR, and CMV disease, meaning tissue damage and symptomatic clinical manifestations, should be made [5]. CMV disease frequently occurs in immunocompromised patients including transplant recipients, patients with acquired immunodeficiency syndrome (AIDS), and patients receiving chemotherapy or steroids [1]. Patients with inflammatory bowel disease (IBD) are often treated with immunosuppressive agents which increase the risk of CMV disease [6].

CMV infection should be considered in IBD patients that present with fever, leukopenia, lymphadenopathy and splenomegaly [7]. However, CMV colitis need not have these features. There are no characteristic endoscopic features that are diagnostic of CMV colitis [8]. CMV infection in existing IBD often worsens the colitis. Whether CMV is a pathogen or an ‘innocent bystander’ representing a reactivation of a latent virus due to immunosuppressive therapy is still unclear. A few studies have shown the spontaneous disappearance of the virus and the limited impact that antiviral therapy has on the course of IBD [9, 10]. Other studies have reported that CMV colitis superimposed on IBD increases the prevalence of toxic megacolon and of surgical intervention risks [11, 12]. Some case reports and series have also shown that patients with severe UC unresponsive to immunosuppressive agents, improve after antiviral treatment for CMV colitis [13, 14].

This study assessed the prevalence and the clinical features of CMV disease in hospitalized IBD patients in a medical center in Taiwan, evaluated the severity of CMV colitis by histology and its impact on IBD patients, searched the possible associated factors, and compared the outcomes between IBD patients with and without CMV colitis.

## Methods

The institutional review board of the National Taiwan University Hospital (NTUH) ethics committee approved this study. This retrospective study reviewed the IBD patients admitted to NTUH, a referral medical center in Taiwan, from January 1, 1998 through December 31, 2013. The patients were identified from the computerized databases of the NTUH using the International Classification of Disease (2001 version) for disease coding (ulcerative colitis (UC) as 556, Crohn’s disease (CD) as 555, respectively). All data were completely reviewed by the same gastroenterologist.

The definition and criteria for UC and CD diagnosis included the combination of clinical, endoscopic, and histological features and the exclusion of an infectious etiology. The definition of a clinical flare up is one requiring admission and retreatment with steroids or the need for new medications to control disease activity. Colitis was classified as proctitis (E1), left sided (E2), and extensive (E3) in UC [15] and ileal (L1), colonic (L2) and ileocolonic (L3) in CD [16] according to the Montreal classifications. The severity of ulcerative colitis was classified according to the Truelove and Witts severity index.

In the NTUH, the clinical practice for treating admitted UC patients due to aggravated symptoms was an initial stool culture to exclude infection. If there was no evidence of infection, intravenous steroids were used as the first line of acute stage management. If the condition did not improve, a further colonoscopy was performed to exclude other causes of symptom aggravation and a biopsy was performed when possible and necessary at that time.

Clinical data including demographic data, clinical symptoms, treatment regiments before CMV diagnosis, history of operation and the disease course of patients were assessed. The clinical outcome of these patients was reviewed after hospital discharge. The definition of a flare-up is aggravated symptoms that require augmented treatment to control said symptoms. According to the European guidelines on UC management, steroid-refractory colitis is defined as patients who have an active disease despite prednisolone up to 0.75 mg/kg/day over a 4 week period [17].

In our study, CMV colitis is defined as having at least one inclusion body detected by haematoxylin and eosin (H&E) or immunohistochemistry staining in 10 to 20 high-power fields of the colorectal mucosa. A latent CMV infection is the carrying of the CMV genome without having active replication or manifestations of clinical symptoms [18]. Four to six biopsies were taken from the ulcerative area when there was a disease flare-up.

A single, experienced pathologist who was blinded to the clinical data reviewed the hematoxylin and eosin staining (H&E) and CMV immunohistochemial (IHC) stained slides of the study period. Immunohistochemical studies were performed with monoclonal anti-CMV antibodies (clone CCH2 and DDG9; Dako) in a single section followed by staining with an Avidin-Biotin complex (ABC) using standard techniques [19]. The number of CMV-positive cells was expressed using a semiquantitative scale: rare, easy to find, or numerous [20, 21].

The clinical condition guided the physician’s decision on whether to administer antiviral drugs. The antiviral therapy in this study consisted of Gancyclovir 250 mg per 12 h and was administered for 2–4 weeks.

### Statistical analysis

The results are expressed as a median and range. The Chi-squared test or two-tailed Fisher exact test were used to compare qualitative variables in IBD patients with a CMV infection. Student’s *t*-test was used for quantitative variables. These analyses were carried out using the SPSS 11.0 program (SPSS, Paris, France). A *p*-value of less than 0.05 was considered significant.

## Results

### Demographic characteristics of patients

During the 15 years from 1998 to 2013, a total of 673 IBD patients were hospitalized. There were 312 patients diagnosed with CD and 361 with UC. Of these patients, 11 (1.6%) were diagnosed with CMV colitis (seven female and four male). The median age of the patients was 47.3 years (range of 21 to 6o years) in UC and 61.3 years (range of 21 to 66 years) in CD. Regarding the patients with IBD, there were no significant differences between the positive and negative CMV colitis groups in gender and operation risk. Among the CD patients, the age differed significantly with an older age observed in the positive CMV group (*p* < 0.005). The demographics and clinical features of patients with the CMV infection are summarized in Table 1.

**Table 1:** Characteristics of IBD patients with or without CMV infection

Variables	Ulcerative colitis - CMV (-)	Ulcerative colitis - CMV (+)	*p*-value	Crohn’s disease - CMV (-)	Crohn’s disease - CMV (+)	*p*-value
Number	356	5		307	6	
Gender (Female)	141 (39.6)	3 (60)	*p* = 0.315	114 (37.1)	4 (66.7)	*p* = 0.205
Age	46 (10–62)	47.3 (21–60)	*p* = 0.672	43 (22–51)	61.3 (21–66)	*p* < 0.005
Operation	19 (5.3)	0	*p* = 1	42 (13.7)	2 (33.3)	*p* = 0.203

### Clinical features

The most frequent symptom of CMV colitis was a bloody stool in UC (*n* =5, 100%) and abdominal pain or bloody stool in CD (*n* =4, 66.7%). Other clinical symptoms were fever (40% in UC; 50% in CD) and body weight loss (40% in UC and 16.7% in CD). Two CD patients had either toxic megacolon or lymphadenopathy (16.7%). All UC CMV colitis patients presented as severe disease and left colon colitis (Montreal classification E2). As for CD with CMV colitis, L2 (colon) involvement was the most common (Table 2).

**Table 2:** Clinical features of CMV colitis in IBD patients

Variables	Ulcerative colitis *n* = 5 (%)		Crohn’s disease *n* = 6 (%)
Symptoms			
Fever	2 (40)		3 (50)
Abdominal pain	3 (60)		4 (66.7)
Lymphadenopathy	0 (0)		1 (16.7)
Bloody stool	5 (100)		4 (66.7)
Body weight loss	2 (40)		0 (0)
Toxic megacolon	0 (0)		1 (16.7)
Location			
Ulcerative procitis (E1)	0 (0)	Ileum (L1)	1 (16.7)
Left side (E2)	5 (100)	Colon (L2)	3 (50)
Extensive (E3)	0 (0)	Ileocecal (L3)	2 (33.3)
Severity			
Mild	0		
Moderate	0		
Severe	5 (100)		

Medications used at the index hospitalization of CMV colitis diagnosis included mesalazine, steroids, azathioprine and anti-TNF α. They were 100, 80, 40 and 0% in UC patients, and 100, 83.3, 66.7 and 33.3% in CD patients, respectively (Table 3). All the 11 CMV colitis patients received mesalazine and three of them received steroids. Four patients received steroids and azathioprine, and two of them received steroids and azathioprine combined with anti-TNF. Among the nine patients who received steroids, 44.4% (4/9) were steroid-refractory colitis. We then conducted a matched case-control analysis. Three controls per each CMV positive case were randomly selected and matched by age and sex from the hospitalized IBD population. We observed that the steroid usage rate was 81.8% in the CMV positive group and 51.5% in the CMV negative group (*p* < 0.005). The azathioprine usage rate was also higher in the CMV positive group (54.5%) compared to the CMV negative patients (21.9%) (*p* = 0.033). There was no significant colectomy rate increase in CMV positive patients (Table 4).

**Table 3:** Medication usage in IBD patients with CMV colitis

	5 ASA	Steroid	Azathioprine	Anti-TNF α	Others
CD	6 (100%)	5 (83.3%)	4 (66.7%)	2 (33.3%)	
CD case 1	+	+	+	+	
CD case 2	+	+	+	-	
CD case 3	+	+	-	-	
CD case 4	+	+	+	-	
CD case 5	+	+	+	+	MTX
CD case 6	+	-	-	-	
UC	5 (100%)	4 (80%)	2 (40%)	0 (%)	
UC case 1	+	+	+	-	Cyclosporin
UC case 2	+	+	+	-	
UC case 3	+	-	-	-	
UC case 4	+	+	-	-	
UC case 5	+	+	-	-

**Table 4:** Medication usage in IBD patients with or without CMV colitis

	CMV positive	CMV negative	*p*-value
Total Number	11	33	
5 ASA	11 (100%)	32 (97%)	*p* = 0.244
Steroid	9 (81.8%)	17 (51.5%)	*p* < 0.005
Azathioprine	6 (54.5%)	7 (21.9%)	*p* = 0.033
Anti-TNF α	2 (18.2%)	3 (9%)	*p* = 0.126

### Treatment and outcomes of CMV colitis

Antiviral therapy was prescribed in 8 of the 11 patients (72.7%), two of them (2/8, 25%) suffered from disease flare-ups and six of them (6/8, 75%) did not have flare-ups in the following year. Three patients did not receive anti-viral treatment since they clinically improved after tapering their steroid treatment. All three of these patients (3/3, 100%) had colitis flare-ups within the 1 year follow up. Two CD patients (33.3%) underwent operations. One of them had a low anterior resection for toxic megacolon in the CMV treated group, and the other one underwent a colectomy for luminal stenosis related partial obstruction in the CMV non-treated group (Table 5).

**Table 5:** Clinical course of CMV colitis in IBD patients

Variables	Ulcerative colitis *n* = 5 (%)	Crohn’s disease *n* = 6 (%)
Treatment		
Gancyclovir > 14 days	4 (80)	4(66.7)
No treatment	1 (20)	2 (33.3)
Outcome		
Colectomy	0 (0)	2 (33.3)
Flare-up	2 (40)	3 (50)

### Histology grading of CMV colitis

**Fig. 1:**
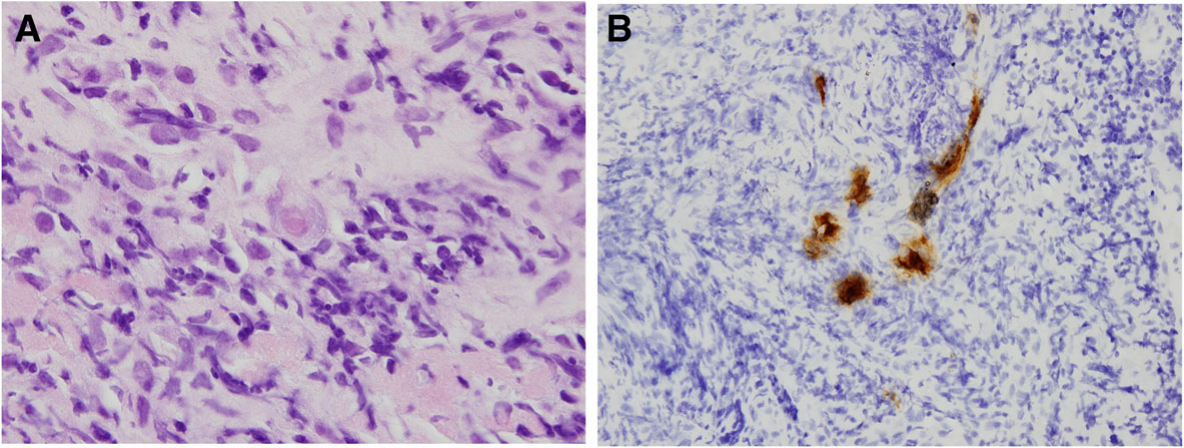
**a** Cytomegalovirus in an endothelial cell (hematoxylin and eosin stain, 1000× magnification). **b** Immunohistochemistry of cytomegalovirus-infected cells (400× magnification)

Positive CMV inclusion bodies in H&E staining (Fig. 1a) and IHC staining (Fig. 1b) were identified in the CMV colitis biopsy specimens. As shown in Table 6, most of the CMV colitis was graded as “rare” by the pathologist. ICH staining was performed in 9 of the 11 and all showed positive results. The cytomegalic cells were usually detected in the granulation tissue of the ulcer. The grade of CMV inclusion didn’t correlate with clinical parameters or to the course of IBD flare ups. From those with flare-ups after the index admission, we found that without antiviral treatment, colitis recurred even with rare CMV inclusion bodies.

**Table 6:** Grades of Cytomegalovirus inclusion and outcome

	CMV inclusion	IHC	Cell type	Granulation	Anti-viral treatment	Flare-up	Operation
C. D							
1	Rare	Yes	Endothelial	Granulation	No	Yes	No
2	Rare	Yes	Endothelial	Granulation	Yes	No	No
3	Rare	Yes	Endothelial	Granulation	Yes	No	No
4	Easy	Yes	Endothelial	Granulation	No	Yes	Yes
5	Rare	No	Endothelial	Granulation	Yes	Yes	Yes
6	Numerous	Yes	Endothelial	Granulation	Yes	No	No
U. C							
1	Rare	Yes	Endothelial	Granulation	Yes	Yes	No
2	Easy	Yes	Endothelial	Granulation	Yes	No	No
3	Rare	No	Endothelial	Granulation	Yes	No	No
4	Rare	Yes	Endothelial	Granulation	Yes	No	No
5	Rare	Yes	Endothelial	(-)	No	Yes	No

## Discussion

The prevalence of tissue HE or IHC positive CMV colitis in IBD patients ranges from 2 to 29% [22]. The known risk factors of CMV colitis in hospitalized patients for IBD exacerbation include 30 or more years of age, disease duration of less than 5 years and immunosuppressive therapy [23]. In our study, CD patients with CMV colitis were more frequently detected in elderly patients. This finding is compatible with a study showing that IBD patients with CMV infection are older [24]. Ten patients in our study had disease duration of less than 5 years. Another previous study analyzed 114 patients with active UC requiring intravenous steroids, steroid-refractory UC, inactive UC and healthy controls. Six CMV positive patients were detected in the steroid–refractory group (30%) but none of the CMV-positive patients responded to steroids in the inactive UC or control groups [25]. Most of the patients in the previous study (5/6) presented with extensive colitis (E3), whereas in our study the CMV positive patients (5/5) were all observed in the left side colon (E2). We also observed higher steroid and azathioprine usage rates in the CMV positive group than in the CMV negative group. One meta-analysis study has revealed a double risk of steroid resistance in CMV-positive IBD patients compared with CMV-negative IBD patients [26].

Endoscopic findings of CMV colitis include a variety of mucosal defects, punched-out ulceration, longitudinal ulceration, and cobblestone-like appearance [25]. Biopsies at the center of the ulcer beds can increase the diagnostic rate [27]. A histological examination is often considered the “gold standard” for CMV colitis diagnosis. Conventional H&E stains are very specific but have low sensitivity, ranging from 10 to 87%, as inclusion bodies are not easily detected and false-negative biopsies are common [28, 29]. One study has shown that 37.5% of biopsies fail to identify any inclusions in CMV colitis [30]. Immunohistochemistry (IHC) with monoclonal antibodies against CMV significantly increases the sensitivity of CMV detection to 78–93% [12, 31]. CMV disease can also be detected by quantitative real-time PCR. Tissue PCR has the highest detection rate compared to CMV antigenemia, H&E and IHC stain [25, 32]. Cases with positive viral DNA without any histological features of CMV may represent latent CMV [29].

Steroid-refractory colitis is also a risk factor for CMV infection [33]. CMV detection by IHC has been reported in 20–40% of patients with severe and/or steroid-refractory colitis and only 0–2.5% in patients with non-refractory colitis [12, 25, 34, 35]. There were 44.4% CMV positive patients with steroid-refractory colitis observed in our study. For all IBD patients, one study detected ten cases of CMV infection in 1,895 IBD patients over a 6-year period with a prevalence of 0.53% [36]. Nine of the ten patients had cytomegalic cells in colon tissue and the remaining one had characteristic inclusion bodies on bronchoscopic biopsy, positive viral culture and CMV IgM. In another retrospective analysis of 1023 IBD patients, the prevalence of CMV disease detected by an IHC stain was 1.37% [37]. In our study, the prevalence of CMV infection in hospitalized IBD patients was 1.6%. The denominator of these two studies was the “total number of patients diagnosed with IBD”, whereas most of the patients did not receive a routine CMV H&E or IHC staining in these three studies which might have led to underestimating the prevalence.

Several studies have investigated CMV’s pathogenic role in IBD. One theory hypothesizes that CMV is an innocent bystander in IBD. This is supported by an in vitro study that shows proliferating cells in granulation tissue are more susceptible to CMV infection [38]. Some clinical studies have not observed any significant differences in disease duration, disease extent or operation rates between severe UC patients with or without a CMV infection [9, 25, 39, 40], whereas other studies revealed that patients with CMV colitis more often have higher operation rates and fatal outcomes [41–43]. In our study, the colectomy rate was 18.2% in the CMV positive group and 9% in the CMV negative group. However, even though this shows a trend, our results did not reach statistical significance, which might be related to the small case number.

The benefits of antiviral therapy in IBD patients with CMV infection are still questionable. Spontaneous disappearance of the virus in steroid responsive IBD patients was reported in one study [35]. Other studies have revealed that antiviral therapy improves the remission rate and surgery-free survival outcomes [34, 44]. One study has suggested that ganciclovir is not required in steroid-responsive patients but is effective in steroid-refractory ulcerative colitis patients [45]. Another study reported that in active UC patients with positive mucosal CMV DNA, the absence of a large ulcer is predictive of latent CMV and requires no antiviral therapy [46]. Currently, antiviral treatment is recommended when CMV is detected in colonic mucosa by the American College of Gastroenterology guidelines [47] and in severe steroid-resistant colitis patients by the European Crohn’s & Colitis Organization (ECCO) guidelines [48]. In our study, all patients without antiviral treatment had colitis flare-ups, even those patients who had a rare IHC grade. Therefore, we suggest treating CMV colitis in hospitalized IBD patients with antiviral agents to decrease the flare-up rate.

## Conclusions

In conclusion, CMV colitis in hospitalized Taiwanese IBD patients is not as common as previously reported in Western countries [22], which could be related to underestimations. Compared to CMV negative IBD patients, CMV colitis patients have more often received steroid and azathioprine treatments. Antiviral treatment is recommended in steroid refractory IBD patients to improve their outcomes.

## Abbreviations

CD: Crohn’s disease
CMV: Cytomegalovirus
IBD: Inflammatory bowel disease
NTUH: National Taiwan University Hospital
UC: Ulcerative colitis
